# A Transmembrane Polymorphism of Fc*γ* Receptor IIb Is Associated with Kidney Deficiency Syndrome in Rheumatoid Arthritis

**DOI:** 10.1155/2016/3214657

**Published:** 2016-03-08

**Authors:** Na Mo, Ruogu Lai, Shizi Luo, Jianglin Xie, Xizi Wang, Lijuan Liu, Xiaoling Liu, Guangxing Chen

**Affiliations:** Division of Rheumatology and Clinical Immunology, The First Affiliated Hospital of Guangzhou University of Chinese Medicine, Guangzhou 510405, China

## Abstract

*Objective.* The purpose is to investigate the role of kidney deficiency and the association between kidney deficiency and a polymorphism Fc*γ*RIIb 695T>C coding for nonsynonymous substitution IIe232Thr (I232T) in rheumatoid arthritis (RA).* Methods.* Clinical parameters and autoantibodies were analyzed and genotyping was performed in 159 kidney deficiency and 161 non-kidney-deficiency RA patients.* Results.* The age of disease onset and disease duration exhibited significant differences between two groups (*P* < 0.01). Patients with kidney deficiency tend to have higher activity of disease (*P* < 0.05). Anti-cyclic citrullinated peptides antibodies (ACPA) levels of patients with kidney deficiency were higher than the controls (*P* = 0.039). 125 (78.6%) kidney deficiency and 114 (70.8%) non-kidney-deficiency patients had both ACPA-positive and RF-positive (*P* = 0.04, OR = 3.29). Fc*γ*RIIb I232TT homozygotes were identified in 10 of 159 (6.3%) kidney deficiency subjects and 1 of 161 (0.6%) controls (*P* = 0.000, OR = 16.45). Furthermore, in pooled genotype analysis, I232IT and I232TT homozygotes were significantly enriched in kidney deficiency individuals compared with the controls (*P* = 0.000, OR = 3.79). Frequency of T allele was associated with kidney deficiency RA population (*P* = 0.000, OR = 3.18).* Conclusion.* This study confirmed that kidney deficiency was closely associated with disease activity and autoimmune disorder in RA. Kidney deficiency in RA is first to reveal a strong genetic link to Fc*γ*RIIb variants.

## 1. Introduction

Rheumatoid arthritis (RA) is an autoimmune disease characterized by chronic inflammation of joints and destruction of cartilage and bone. Although the precise etiology of RA has been unclear, genetic deficiency and autoimmune disorder are generally considered as contribution to RA. In the underlying mechanisms of RA, B cells were regarded to have a fundamental role in the pathogenesis of RA for a variety of autoantibodies produced in RA patients including rheumatoid factors (RF), type II collagen antibodies, and anti-cyclic citrullinated peptides antibodies (ACPA) [1–3]. In thresholds of keeping autoimmune tolerance, Fc gamma receptor b (Fc*γ*RIIb) is the major receptor that inhibits coreceptor of B cell receptor (BCR) to reduce the risk of autoimmune diseases. However, a polymorphism Fc*γ*RIIb 695T>C codes for nonsynonymous substitution IIe232Thr (I232T) in the transmembrane segment of the receptor and amino acid change influences receptor-mediated functions [4]. Defective Fc*γ*RIIb polymorphism is associated with Systemic Lupus Erythematosus (SLE) and joints damage of RA [5, 6].

In Traditional Chinese Medicine (TCM), deficiency of kidney is a critical factor in etiology and pathogenesis of RA which modern biological mechanism does not understand. Kidney is regarded as generating marrow and dominating bone according to Visceral Manifestation theory of TCM. For B cells glow in bone marrow, we supposed that B cells might be correlated with kidney. RA kidney deficiency would result in defective B cells. Our previous study showed that the risk of autoantibodies like antinuclear antibody (ANA) and RF in RA patients with deficiency of kidney is more than non-kidney-deficiency patients. Kidney deficiency was suggested to be associated with Fc*γ*RIIb downregulation [7, 8]. However, the mechanism of Fc*γ*RIIb reduction is still unknown.

Kidney is often considered as the congenital foundation and the origination of essence in TCM theory. We presumed that kidney deficiency could relate to congenitally deficiency or genetic defect, which leads to breakdown of immune tolerance in autoimmune diseases like RA. In addition, kidney generates marrow and dominates bone function. Kidney deficiency might have effect on disease activity and bone damage of RA. But there was just a hypothesis of TCM theory in the absence of support by scientific researches. So it is important to investigate the role of kidney deficiency on RA and the association between kidney deficiency and genetic susceptibility based on massive cases.

Here, our results showed that patients with kidney deficiency experienced high disease activity, levels of ACPA, and proportion of ACPA-positive and RF-positive regarded as reliable predictors of radiographic progression RA. Fc*γ*RIIb I232T polymorphism presented a strong genetic link to kidney deficiency RA.

## 2. Materials and Methods

### 2.1. Study Design and Subjects

This study recruited 320 Chinese RA patients (159 kidney deficiency subjects and 161 non-kidney-deficiency subjects) from Jun 2011 to Feb 2015 at Department of Rheumatology and Clinical Immunology, The First Affiliated Hospital of Guangzhou University of Chinese Medicine (TCM) (Guangzhou, China). The local ethics committee of The First Affiliated Hospital of Guangzhou University of TCM approved the protocol of this research and all patients wrote informed consent for the research. Every subject was diagnosed by rheumatology specialists according to the 2010 American Rheumatism Association criteria for RA exclusion of other autoimmune diseases, including heart failure, kidney failure, and tumor diseases [9]. Patient less than 16 years or older than 80 years was excluded. All clinical parameters were collected including gender, age at disease onset, disease duration, and swollen and tender joints counts.

### 2.2. Judgment of Kidney Deficiency

Specialists of TCM judged kidney deficiency based on the 1986 deficiency syndrome of criteria of TCM by Integrative Medicine Deficiency Syndrome and Senile Diseases Research Association and the 1994 disease and classification criteria of diagnosis and treatment effect by State Administration of TCM of China [10, 11].

*Inclusion Criteria*. Sore lower back (exclude injury); tibial aching and limp or heel pain; limited bending forward and backward; alopecia or teeth shaking; dribble of urine or incontinence; sexual dysfunction or infertility; fear of cold and cold limbs; fatigue. The classification of kidney deficiency in RA is applied to any individual who meets inclusion criteria equal to or more than 5 items.

### 2.3. Disease Activity

ESR was detected by the Westergren method and CRP was measured by nephelometric method. Disease activity of every subject was assessed using DAS28 of The European League Against Rheumatism (EULAR). All RA patients were categorized as low activity and remission, moderate activity, and high activity. Low activity and remission were defined as DAS28 ≤ 3.2. Patients were considered to have middle active disease as 3.2 < DAS28 ≤ 5.1. High active disease was DAS28 > 5.1.

### 2.4. Autoantibodies Measure

The levels of RF were tested by nephelometric assay and ACPA were analyzed by chemiluminescence assay: RF > 20 IU/mL and ACPA > 5 U/mL were considered positive. Double positive of autoantibodies was defined as both RF-positive and ACPA-positive.

### 2.5. Genotyping of Fc*γ*RIIB-232I/T Using Genomic DNA

Peripheral bloods of RA patients were obtained. Because amino acid and nucleotide sequences of Fc*γ*RIIb are highly homologous to FcgRIIA and IIC in exons 1–5, the nested polymerase chain reaction (PCR) was employed to avoid the variations deriving from FCGR2A or FCGR2C polymorphisms. The Beijing Genomics Institute (BGI) performed DNA exacting, nested PCR, and sequencing.

Genomic DNA was exacted using the Promega DNA isolation kit (Promega Corporation, Madison, USA). For amplification of fragments, nested PCR was performed as described by Kyogoku et al. [12]. Firstly, PCR amplified a 4,323-bp fragment of Fc*γ*RIIb in a 50 *μ*L volume using the Expand Long Template PCR System (BIO-RAD) extending from exon 4 to exon 7 (forward primer 5′-AAGGACAAGCCTCTGGTCAA-3′; reverse primer 5′-CCCAACTTTGTCAGCCTCAT-3′). PCR was performed in a 50 *μ*L volume containing 3 *μ*L cDNA (ng/*μ*L), 5 *μ*L 10x Ex Taq buffer, 4 *μ*L dNTP (2.5 mM each), 3 *μ*L primer each, 0.25 *μ*L Ex Taq (5 U/*μ*L), and 31.75 *μ*L H_2_O. Amplification was carried out with conditions as follow: initial denaturation at 96°C for 5 minutes, 30 cycles of denaturation at 96°C for 20 seconds, annealing at 55°C for 30 seconds, and extension at 72°C for 1 minutes, followed by a final extension at 72°C for 5 minutes. After the fragment was checked, a nested PCR followed (forward primer 5′-TGTGACCATCACTGTCCAAG-3′; reverse primer 5′-CTGAAATCCGCTTTTTCCTG-3′). the nested PCR was carried out in a 30 *μ*L volume containing a 50 *μ*L volume containing 2 *μ*L cDNA (ng/*μ*L), 3 *μ*L 10x Ex Taq buffer, 2 *μ*L dNTP (2.5 mM each), 1 *μ*L primer each, 0.2 *μ*L Ex Taq (5 U/*μ*L), and 20.8 *μ*L H_2_O. Conditions of amplification were as follows: initial denaturation at 96°C for 5 minutes, 30 cycles of denaturation at 96°C for 20 seconds, annealing at 54.8°C for 30 seconds, and extension at 72°C for 1 minutes, followed by a final extension at 72°C for 5 minutes. To distinguish between FCGR2B and FCGR2C, a nested-over-nested PCR was performed on the purified 863-bp fragment from carriers of the variant allele (diluted 10,000x) (forward primer 5′-GGGAGCCCTTCCCTCTGT-3′; reverse primer 5′-GGAGGCATAAGTCCAGCCAC-3′). PCR conditions were similar to those in the nested PCR (annealing at 64°C). Finally, the 395-bp production was sequencing directly (primer 5′-GGAGGCATAAGTCCAGCCAC-3′).

### 2.6. Statistical Analysis

All values of analysis were expressed as means and standard error of mean among RA patients according to onset ages, disease duration, tender joint counts, swollen joint counts, ESR, CRP, DAS28 scores, and levels of RF and ACPA. Chi-square analyses were used to analyze the difference between two groups of RA patients. Distribution of DAS28 scores, both RF-positive and ACPA-positive, and frequencies of Fc*γ*RIIb genotypes and allele were tested with chi-square tests suing the SPSS18 statistics package for Windows (SPSS, Chicago, IL, USA). *P* values of less than or equal to 0.05 were considered statistically significant.

## 3. Results

### 3.1. Demographic and Clinical Characteristics of Chinese RA with Kidney Deficiency and No Kidney Deficiency

In order to establish study cohort, 320 RA patients (251 females and 69 male) were recruited. The youngest patient was 10 years old at age of disease onset while the oldest subject was 77 years old among 320 RA patients. These subjects had disease duration at the mean RA disease duration of 46.13 ± 14.76 months, from 2 to 684 months. The current study cohort included 159 kidney deficiency and 161 non-kidney-deficiency RA patients. As shown in Table 1, no differences in gender were observed between two groups of RA patients (*P* = 0.314). Patients with kidney deficiency were older compared with non-kidney-deficiency patients at age of disease onset (50.96 ± 13.21 years versus 40.63 ± 13.21 years; *P* = 0.000). The disease duration of kidney deficiency subjects was longer than non-kidney-deficiency individuals (129.08 ± 129.48 months versus 95.31 ± 74.91 months; *P* = 0.005). These data indicated that kidney deficiency was associated with age at disease onset and disease duration of RA patients.

### 3.2. Association of High Disease Activity with Kidney Deficiency in RA Patients

To explore the link of activity of RA disease with kidney deficiency syndrome, we next evaluated the activity of disease based on tender and swollen joint counts, ESR, CRP, and DAS28. As shown in Table 2, kidney deficiency RA patients tend to have higher activity of disease because there was difference between kidney deficiency and non-kidney-deficiency RA patients in tender joint counts, ESR, and DAS28 (*P* = 0.031, *P* = 0.045, and *P* = 0.022 resp.). Differences did not reach statistical significance in swollen joints and CRP (*P* = 0.357 and *P* = 0.67).

Furthermore, we investigated the relationship between kidney deficiency RA and categories of disease activity defined as high, moderate, and low. 71 (44.1%) non-kidney-deficiency patients and 52 (32.7%) kidney deficiency patients had low and moderated disease activity in Table 3. The proportion of kidney deficiency populations with high activity significantly increased compared with non-kidney-deficiency populations (*P* = 0.039; odds ratio 1.62; 95% CI 1.03–2.56). This result suggested that kidney deficiency was associated with high activity of RA.

### 3.3. Autoantibodies Levels of RA Patients with Kidney Deficiency and No Kidney Deficiency

To observe the effect of kidney deficiency on autoimmune disorders of B cells, levels of RF and ACPA are analyzed. The results (shown in Table 4) demonstrated that ACPA levels of patients with kidney deficiency were higher than those in non-kidney-deficiency group (127.50 ± 80.46 versus 107.38 ± 82.37; *P* = 0.039). Kidney deficiency subjects have higher RF titer (685.89 ± 1215.49 versus 524.09 ± 835.39). However, no statistical difference was found between two groups in RF levels (*P* = 0.166).

### 3.4. Kidney Deficiency Is Strongly Associated with Poor Prognosis of Radiographic Progression in RA Population

What is more, the correlation of double RF+/ACPA+ to kidney deficiency was investigated in this cohort study. All patients were divided into three groups: RF−/ACPA−, single positive (RF+ or ACPA+), and double positive (RF+/ACPA+). Compared with RF−/ACPA− individuals, 125 (78.6%) kidney deficiency individuals and 114 (70.8%) non-kidney-deficiency controls had RF+/ACPA+ in Table 5 (*P* = 0.04, OR = 3.29, and 95% CI 1.03–10.49).

### 3.5. Fc*γ*RIIb IIe232Thr Polymorphism Is Associated with Chinese RA Patients with Kidney Deficiency

Importantly, we stratified Fc*γ*RIIb SNP IIe232Thr genotype and allele distributions in RA patients with kidney deficiency.  Table 6 shows the frequencies of Fc*γ*RIIb I232T genotypes in the kidney deficiency and non-kidney-deficiency individuals. The I232II allele positivity reduced and I232IT increased in kidney deficiency patients. In particular, I232TT homozygotes were identified in 10 of 159 (6.3%) kidney deficiency subjects and 1 of 161 (0.6%) non-kidney-deficiency controls. Moreover, I232IT and I232TT homozygotes were significantly enriched in kidney deficiency individuals compared with the controls (*χ* ^2^ = 30.5,  *P* = 0.000, and OR = 3.79 (95% CI 2.34–6.15) by 2 × 2 contingency tables analysis). Finally, 88.51% (285/322) Thr in kidney deficiency RA population was compared with 70.75% (225/318) in the controls. This difference reached statistical significance (*P* = 0.000).

Taken together, these results suggested that I232IT and I232TT were risk factors for RA populations with kidney deficiency.

## 4. Discussion

RA is diagnosed as “BiZhen” or “LiJie disease” in TCM. Kidney deficiency is regarded as a critical determinant to cause and procession in BiZhen or LiJie disease. Wan Kentang found that kidney deficiency was the most important fundamental cause of BiZhen. Now it is generally recognized in TCM that BiZhen might be initiated by wind, wet, cold, and heat of etiology based on kidney deficiency. Congenital deficiency, excessive sexual intercourse, aging, and long disease duration might lead to kidney deficiency [13–15]. According to Huangdi's Canon of Medicine theory, woman's life may be divided into seven phases and each phase lasts for seven years. Women become exhaustion of kidney-essence with diminishing reproductive function to induce to menopause on 7th phase (about 49 years old). The epidemiological research demonstrated a peak age of onset of RA at the time of the menopause in women [16]. Moreover, early age at menopause (≤45 years) was revealed to be a link to the subsequent risk factor of RA [17]. The preceding clinical experimental and prospective epidemiological studies [18] suggested that dysregulation of adrenocortical or gonadal function may predispose to developing RA. Our data showed that the mean age of RA disease onset was 50.96 years in kidney deficiency patients, which is approximate to the phase of kidney-essence exhausting. This study serves as a proof-of-concept that kidney deficiency not only impairs reproductive function but also increases susceptibility to common human autoimmune disorders for neuroendocrine immune (NEI) pathogenesis. What is more, we found that kidney deficiency RA undergoes longer disease procession compared with the controls, which provided an evidence that long disease duration would result in kidney deficiency.

Except for etiology, kidney deficiency is more relevant with disease progression of bone erosion. Deep and weak pulse on cunkou pulse of patients with LiJie disease in* Synopsis Golden Chamber* (one of four TCM classic works) were recorded. Deep pulse on cunkou pulse indicated that kidney deficiency played an important role in bone destruction. Some rheumatologists of TCM like Shude and Liangchun thought that kidney deficiency could induce disorder of the kidney generating marrow and dominating bone function to destroy joints [13, 19]. However, there was still no enough massive clinical evidence to prove kidney deficiency correlation to disease activity and joints destruction previously. In the present study with 320 cases, kidney deficiency RA patients tend to be with higher activity of disease than non-kidney-deficiency individuals on account of tender joints, ESR, DAS28, and proportion of high DAS28 scores. In RA processing of progressive erosion and cartilage destruction, RF and ACPA were considered as reliable predictors of radiographic progression. RA individuals with positive RF or ACPA were categorized to be patients with poor prognosis who would be treated more actively than without poor prognosis [20]. In particular, patients with high titers of ACPA were prone to radiographic progression [21]. In addition, Van Steenbergen et al. found that the presence of both ACPA+/RF− and ACPA+/RF+ was associated with more severe erosive progression than ACPA−/RF− based on evaluating total 1393 RA patients and 6023 sets of radiographs [22]. It is recorded in the theory of TCM Visceral Manifestation that the kidney generates marrow and dominates bone. Once kidney was deficiency, bone would not be nourished to get destruction. Thus, kidney deficiency usually was thought to play an important role in RA progressive bone erosion. Our present results showed that RA patients with kidney deficiency had elevated ACPA titers compared to no kidney deficiency. What is more, we found more proportion of the population with high ACPA levels and both ACPA-positive and RF-positive in kidney deficiency. This data demonstrated that kidney deficiency RA patients would be at higher risk of joints destruction for poor prognosis.

In TCM theory, kidney deficiency is involved in congenital deficiency. Attention was paid to genetic susceptibility to autoimmune diseases increasingly. Functionally defective variants of Fc*γ*RIIb already identified association with ACPA antibody production, RF production, destructive RA, and early onset of RA in Taiwanese [23]. Fc*γ*RIIb, expressing B cells and many myeloid cells, is the major transmembrane receptor providing feedback inhibitory signals for cell activation. B cell receptor (BCR) signaling was initiated by immune complexes. Then, Fc*γ*RIIb coligating with BCR leads to dephosphorylation of CD19 tyrosines, the tyrosine phosphorylation of the immunoreceptor tyrosine-based inhibitory motif (ITIM) in the Fc*γ*RIIb cytoplasmic domain, and the recruitment of SH2-containing inositol 5-phosphatase (SHIP), inhibiting calcium influx. Finally, BCR and downstream signaling cascades are blocked to induce immunologic tolerance [24]. Fc*γ*RIIb gene locates on chromosomes 1q23. Mice with defective Fc*γ*RIIb gene are susceptible to type II collagen induced arthritis with severe joints damage [25]. On the contrary, overexpression of Fc*γ*RIIb on B cells could relieve severity of collagen induced arthritis (CIA) [26]. Moreover, population with defective Fc*γ*RIIb gene would increase risks of bone damage of RA and SLE. Catalan reported that RA patients had a high percentage of naïve and memory B cells expressing CD86. Nevertheless, the expression of Fc*γ*RIIb decreased significantly on RA memory B cells and plasmablasts compared with healthy donors; the downexpression of Fc*γ*RIIb was inversely associated with high levels of anti-citrullinated vimentin (anti-MCV) autoantibodies which correlates with autoantibody levels [27, 28]. Radstake studied the effect of the FCGR2B 695T>C polymorphism on susceptibility to RA, severity of the disease, and DC function. RA patients with Fc*γ*RIIb 695T>C variant had significantly more radiologic joint destruction than those carrying the Fc*γ*RIIb wild-type alleles, which suggested that the Fc*γ*RIIb variant was an independent and by far the strongest predictor of radiologic joint damage by altering its function failed to display the inhibitory [5, 6]. TCM considers kidney deficiency as congenitally deficient partly, which result in susceptibility to autoimmune diseases like SLE and RA. We revealed kidney deficiency RA patients with higher proportion of Fc*γ*RIIb I232IT and I232TT homozygotes than non-kidney-deficiency controls. The odds ratio for inheriting Fc*γ*RIIb I232TT alleles was 16.45 in kidney deficiency RA. Further, the frequency of Thr232 increased in the kidney deficiency RA populations. Our finding is the first to represent a strong genetic link to susceptibility in kidney deficiency RA. Fc*γ*RIIb variant might be an independent predictor of kidney deficiency. These data suggested that Kidney deficiency might produce much more autoantibodies like RF and ACPA, and a higher proportion of autoreactive B cells result in autoimmune disorders and bone damage by the defective Fc*γ*RIIb function.

In summary, our present study confirmed that kidney deficiency was closely associated with disease activity and autoimmune disorder in RA. Kidney deficiency could be regarded as a poor prognosis of radiographic progression RA for correlation with ACPA-positive and double positive (RF+/ACPA+). Most importantly, these findings revealed a strong genetic association between Fc*γ*RIIb variants and kidney deficiency RA, which is helpful to perform further in-depth research to explain a novel biological mechanism of kidney deficiency.

## Figures and Tables

**Table 1:** Demographic and clinical characteristics of RA.

	No kidney deficiency	Kidney deficiency
Male/female	31 (19.25%)/130 (80.75%)	38 (23.90%)/121 (76.10%)
Age at disease onset (years)	40.63 ± 13.21	50.96 ± 13.21^*∗∗*^
Disease duration (months)	95.31 ± 74.91	129.08 ± 129.48^*∗∗*^

Data are means (SD).

^*∗∗*^Significant (*P* < 0.01) difference between RA patients with kidney deficiency and no kidney deficiency.

**Table 2:** Characteristics of disease activity in RA patients.

	No kidney deficiency	Kidney deficiency
Tender joint counts (0–28 scale)	9.26 ± 8.66	11.42 ± 9.15^*∗*^
Swollen joint counts (0–28 scale)	8.25 ± 7.72	9.09 ± 8.54
ESR (mm/h)	58.66 ± 36.42	66.81 ± 35.76^*∗*^
C-reactive protein (mg/L)	38.41 ± 41.34	40.49 ± 44.15
DAS28 (ESR) scores	5.33 ± 1.54	5.73 ± 1.58^*∗*^

Data are means (SD).

^*∗*^Significant (*P* < 0.05) difference between RA patients with kidney deficiency and no kidney deficiency.

**Table 3:** Association of categories of RA disease activity with kidney deficiency.

Disease activity	No kidney deficiency	Kidney deficiency	*χ* ^2^	*P*	OR	95% CI
Low (≤3.2)	17 (10.6%)	12 (7.5%)				
Moderate (≤5.1)	54 (33.5%)	40 (25.2%)				
High (>5.1)	90 (55.9%)	107 (67.3%)				
Pooled analysis						
Low and moderate	71 (44.1%)	52 (32.7%)				
High (>5.1)	90 (55.9%)	107 (67.3%)	4.39	0.039	1.62	1.03–2.56

The *χ* ^2^ test was used to calculate the *P* values. The odds ratio (OR) and 95% confidence interval (95% CI) were calculated by pooled analysis of low and moderate and high disease activity between kidney deficiency and no kidney deficiency.

**Table 4:** Levels of rheumatoid factor and ACPA in RA patients with kidney deficiency and no kidney deficiency.

Autoantibodies	No kidney deficiency	Kidney deficiency
RF	524.09 ± 835.39	685.89 ± 1215.49
ACPA	107.38 ± 82.37	127.50 ± 80.46^*∗*^

Data are means (SD).

^*∗*^Significant (*P* < 0.05) difference between RA patients with kidney deficiency and no kidney deficiency.

**Table 5:** Association of double positive of autoantibodies with kidney deficiency.

Positive of autoantibodies	No kidney deficiency	Kidney deficiency	*χ* ^2^	*P*	OR	95% CI
RF−/ACPA−	12 (7.5%)	4 (2.5%)				
Single positive	35 (21.7%)	30 (18.9%)				
RF+/ACPA+	114 (70.8%)	125 (78.6%)	4.47	0.04^*∗*^	3.29	1.03–10.49

^*∗*^Compared with double negative group.

**Table 6:** Frequency of the Fc*γ*RIIb IIe232Thr polymorphism in patients with kidney deficiency and no kidney deficiency.

Genotype frequency	Kidney deficiency	No kidney deficiency	*χ* ^2^	*P*	OR	95% CI
Individual genotype						
I232II	76 (47.8%)	125 (77.6%)			1	
I232IT	73 (45.9%)	35 (21.7%)	24.96	0.000^†^	3.43	2.09–5.62
I232TT	10 (6.3%)	1 (0.6%)	12.20	0.000^†^	16.45	2.06–131.04
Pooled genotype analysis						
I232II	76 (47.8%)	125 (77.6%)				
I232IT + I232TT	83 (52.2%)	36 (22.3%)	30.50	0.000^‡^	3.79	2.34–6.15
Allele frequency						
T	285 (88.51%)	225 (70.75%)	31.16	0.000^‡^	3.18	2.09–4.84
I	37 (11.49%)	93 (29.25%)				

^†^By chi-square test with 3 × 2 contingency tables analysis (2 degrees of freedom (df)).

^‡^Calculated using the chi-square test for 2 × 2 contingency tables analysis (df).

The odds ratio (OR) and 95% confidence interval (95% CI) were calculated in comparison with the wild type.
